# Missense substitutions at a conserved 14-3-3 binding site in HDAC4 cause a novel intellectual disability syndrome

**DOI:** 10.1016/j.xhgg.2020.100015

**Published:** 2020-11-21

**Authors:** Emma Wakeling, Meriel McEntagart, Michael Bruccoleri, Charles Shaw-Smith, Karen L. Stals, Matthew Wakeling, Angela Barnicoat, Clare Beesley, DDD Study, Andrea K. Hanson-Kahn, Mary Kukolich, David A. Stevenson, Philippe M. Campeau, Sian Ellard, Sarah H. Elsea, Xiang-Jiao Yang, Richard C. Caswell

**Affiliations:** 1 North East Thames Regional Genetics Service, Great Ormond Street Hospital for Children NHS Foundation Trust, Great Ormond Street, London WC1N 3JH, UK; 2 Medical Genetics, Floor 0 Jenner Wing, St George’s University Hospitals NHS Foundation Trust, Cranmer Terrace, London SW17 0RE, UK; 3 Rosalind and Morris Goodman Cancer Research Centre, McGill University, Montreal, Quebec, QC H3A 1A3, Canada; 4 Department of Medicine, McGill University Health Center, Montreal, Quebec, QC H3A 1A3, Canada; 5 Department of Clinical Genetics, Royal Devon and Exeter NHS Foundation Trust, Exeter EX1 2ED, UK; 6 Exeter Genomics Laboratory, Royal Devon and Exeter NHS Foundation Trust, Exeter EX2 5DW, UK; 7 Institute of Biomedical and Clinical Science, University of Exeter Medical School, Exeter EX2 5DW, UK; 8 Rare & Inherited Disease Laboratory, North Thames Genomic Laboratory Hub, Great Ormond Street Hospital for Children NHS Foundation Trust, 37 Queen Square, London WC1N 3BH, UK; 9 Deciphering Developmental Disorders, Wellcome Genome Campus, Hinxton, Cambridge CB10 1SA, UK; 10 Department of Genetics, Stanford University School of Medicine, 300 Pasteur Drive, H315, Stanford, CA 94305-5208, USA; 11 Department of Pediatrics, Division of Medical Genetics, Stanford University, 300 Pasteur Drive, H315, Stanford, CA 94305-5208, USA; 12 Clinical Genetics, Cook Children’s Medical Center, Fort Worth, TX 76104, USA; 13 Department of Pediatrics, CHU Sainte-Justine Hospital, University of Montreal, Montreal, Quebec, QC H3T 1C4, Canada; 14 Department of Molecular and Human Genetics, Baylor College of Medicine, Houston, TX 77030, USA; 15 Department of Biochemistry, McGill University Health Center, Montreal, Quebec, QC, Canada

**Keywords:** Histone deacetylase 4, intellectual disability, gain-of-function, 14-3-3 binding

## Abstract

Histone deacetylases play crucial roles in the regulation of chromatin structure and gene expression in the eukaryotic cell, and disruption of their activity causes a wide range of developmental disorders in humans. Loss-of-function alleles of *HDAC4*, a founding member of the class IIa deacetylases, have been reported in brachydactyly-mental retardation syndrome (BDMR). However, while disruption of HDAC4 activity and deregulation of its downstream targets may contribute to the BDMR phenotype, loss of HDAC4 function usually occurs as part of larger deletions of chromosome 2q37; BDMR is also known as chromosome 2q37 deletion syndrome, and the precise role of HDAC4 within the phenotype remains uncertain. Thus, identification of missense variants should shed new light on the role of HDAC4 in normal development. Here, we report seven unrelated individuals with a phenotype distinct from that of BDMR, all of whom have heterozygous *de novo* missense variants that affect a major regulatory site of HDAC4, required for signal-dependent 14-3-3 binding and nucleocytoplasmic shuttling. Two individuals possess variants altering Thr244 or Glu247, whereas the remaining five all carry variants altering Pro248, a key residue for 14-3-3 binding. We propose that the variants in all seven individuals impair 14-3-3 binding (as confirmed for the first two variants by immunoprecipitation assays), thereby identifying deregulation of HDAC4 as a pathological mechanism in a previously uncharacterized developmental disorder.

## Teaser

Loss-of-function variants in histone deacetylase 4 (HDAC4) have been reported to cause brachydactyly-mental retardation (BDMR) syndrome through haploinsufficiency. This report describes 7 individuals with a phenotype distinct from BDMR, all with missense variants in a conserved 14-3-3 site, which are hypothesized to result in a novel gain-of-function effect in HDAC4.

## Introduction

Histone deacetylases (HDACs) are crucial regulators of chromatin structure and gene expression throughout the lifetime of the eukaryotic cell.^1^, ^2^, ^3^ In humans, a total of 18 HDACs have been identified, which are divided into four classes: class I (HDAC1, -2, -3, and -8) and II (HDAC4, -5, -6, -7, -9, and -10) enzymes share homology with the related yeast proteins Rpd3 and Hda1, respectively; class III enzymes are represented by five human sirtuins (SIRT1-5), related to yeast Sir2; and class IV is represented by a single enzyme, HDAC11, which shows homology to the catalytic domain of both class I and II enzymes.^1^^,^^2^^,^^4^ Class II enzymes are further subdivided on the basis of their domain architecture: class IIa enzymes (HDAC4, -5, -7, and -9) have an N-terminal regulatory domain and shuttle between the cytoplasm and nucleus, whereas class IIb enzymes (HDAC6 and -10) lack this regulatory region.^1^^,^^2^ The role of class I enzymes in deacetylation of histones within core nucleosomes is well established; however, the activity of class II HDACs toward these targets remains debatable,^1^^,^^2^ and HDAC4 and its three paralogs are now believed to function predominantly as transcriptional co-repressors.^4^, ^5^, ^6^, ^7^, ^8^, ^9^ HDAC4 interacts with, and represses the activity of, transcription factors such as Myocyte Enhancer Factor 2C (MEF2C) and Runt-Related Transcription Factor 2 (RUNX2), which are essential components in various developmental processes.^6^, ^7^, ^8^ Consistent with this, cell-based differentiation assays *in vitro* and mouse genetic studies *in vivo* have shown HDAC4 to be important for development of muscle, bone, brain, and mammary epithelia,^10^, ^11^, ^12^ suggesting a wide role in normal human development and a requirement for the tight regulation of HDAC4 activity.

In support of an important role in development, heterozygous loss-of-function variants in *HDAC4* have been reported to cause brachydactyly-mental retardation syndrome (BDMR [MIM: 600430]),^13^, ^14^, ^15^, ^16^ common features of which are brachydactyly type E, mild to moderate intellectual disability (ID), seizures, autism spectrum disorder, short stature, obesity, and facial dysmorphism. However, the majority of these variants occur as part of larger deletions of chromosome 2q37; hence, the phenotype is also known as chromosome 2q37 deletion syndrome. As a result, there is some doubt as to which features of the phenotype are specific to loss of HDAC4 function per se and which occur as part of a contiguous gene syndrome, a situation that may be further confounded by incomplete penetrance of the *HDAC4* haploinsufficiency phenotype.^14^, ^15^, ^16^ It is also currently unclear as to whether missense variants in *HDAC4* can cause BDMR, although the gene is intolerant of loss-of-function variants, as indicated by a value of 1 for the Genome Aggregation Database (gnomAD) constraint metric for loss-of-function intolerance, pLI;^17^ thus, it is conceivable that variants that significantly impair the catalytic, co-repressor, or other functions of HDAC4 could indeed be pathogenic in the heterozygous state. However, this possibility remains to be investigated.

**Figure 1:**
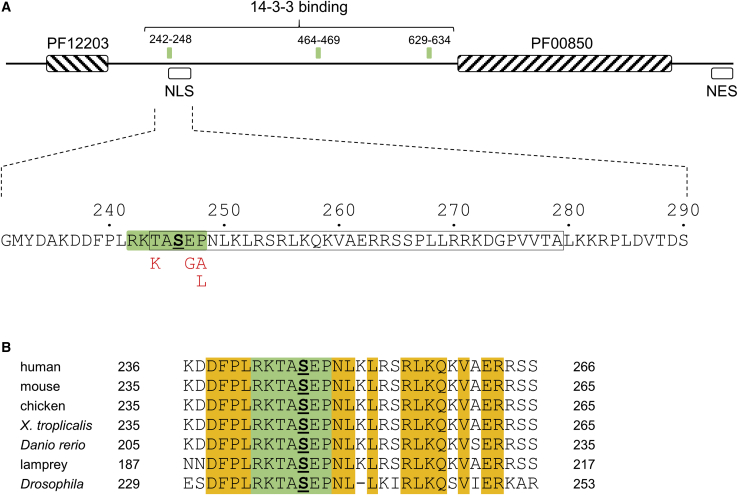
Schematic organization of HDAC4 and location of variants (A) The upper figure shows the organization of the 1,084-residue HDAC4 protein; hatched boxes show Pfam domains PF12203 (glutamine-rich N-terminal domain of HDAC4; residues 62–152) and PF00850 (histone deacetylase domain; residues 675–992) as indicated; green boxes indicate known sites of 14-3-3 binding; open boxes show positions of the nuclear localization signal (NLS) and nuclear export signal (NES). The region around the first 14-3-3 site is shown expanded in the lower figure; the 14-3-3 site spanning residues 242–248 is shaded green, with the phosphorylated serine, Ser246, underlined in bold font; the NLS (244–279) is boxed; missense variants described in this report (p.Thr244Lys, p.Glu247Gly, p.Pro248Ala, and p.Pro248Leu) are shown below the HDAC4 sequence in red font. (B) Residues 236–266 of HDAC4 are shown aligned to orthologs; the core 14-3-3 site, RKTASEP, is shaded green and is invariant in all sequences; other invariant residues are shaded orange. UniProtKB accession codes for HDAC4 orthologs are as follows: human, UniProtKB: P56524; mouse, UniProtKB: Q6NZM9; chicken, UniProtKB: P83038; *Xenopus tropicalis*, UniProtKB: F7CSW6; *Danio rerio*, UniProtKB: Q08BS8; lamprey, UniProtKB: S4RJL9; *Drosophila melanogaster*, UniProtKB: Q9VYF3.

The nuclear activity of HDAC4 is regulated by the process of nucleocytoplasmic shuttling, which in turn is dependent on the phosphorylation-dependent binding of 14-3-3 proteins, which act to sequester HDAC4 in the cytoplasm; as such, HDAC4 serves as a transducer of upstream signaling events.^1^, ^2^, ^3^^,^^10^ *In vitro* studies showed that overexpression of HDAC4 resulted in a predominantly cytoplasmic distribution, with some cells displaying nuclear speckling, and that nuclear accumulation of HDAC4 could be induced either by blocking exportin-1-mediated nuclear export using leptomycin B or by mutation of specific serine residues 246, 467, and 632 to alanine.^18^^,^^19^ These residues all lie within binding sites for 14-3-3 proteins (Figure 1A), with residues 242–248 playing the major role in the regulation of nucleocytoplasmic shuttling. This site partially overlaps the nuclear localization signal in HDAC4, and alone of these three 14-3-3 sites is invariant in HDAC4 orthologs from human to fruit fly (Figure 1B).^2^ Thus, an important question is whether variation at this site, which may result in deregulation of nucleocytoplasmic shuttling, constitutes a pathological mechanism.

Here, we present the cases of seven unrelated individuals with a range of overlapping phenotypic features that are distinct from BDMR. All were found to harbor heterozygous *de novo* missense variants around Ser246, the phosphorylated residue within the key 14-3-3 binding site of HDAC4, and which in the two variants tested were shown to result in reduced 14-3-3 binding. We propose that these variants confer a gain-of-function effect on HDAC4 activity by deregulation of nucleocytoplasmic shuttling.

## Subjects and methods

### Subjects and recruitment

All individuals reported had been referred for investigation of developmental delay (DD) and/or ID of unknown cause and were each assessed clinically by at least one author. Individuals 2–4 were recruited to the Deciphering Developmental Disorders (DDD) Study (see Table 1 for associated DECIPHER accession numbers).^20^ Written informed consent was obtained for genetic studies and publication of all photographs. All studies were approved by ethics boards of the participating institutions in accordance with the Declaration of Helsinki.

**Table 1:** Clinical and molecular features of individuals with *HDAC4* variants at a major 14-3-3 binding site

**Individual #**	**1**	**2**	**3**	**4**	**5**	**6**	**7**
Variant database accession	ClinVar: SCV001427055	DECIPHER: 300920	DECIPHER: 275175	DECIPHER: 286901	ClinVar: SCV000741300.1	ClinVar: SCV000574314.5	ClinVar: SCV001427054
Age	2 years	6 years	20 years	14 years	5 years	5 years	10 years
Gender	female	male	female	male	male	male	female
*HDAC4* variant	NM_006037.4: c.731C>A p.(Thr244Lys)	NM_006037.4: c.740A>G p.(Glu247Gly)	NM_006037.4: c.742C>G p.(Pro248Ala)	NM_006037.4: c.743C>T p.(Pro248Leu)	NM_006037.4: c.743C>T p.(Pro248Leu)	NM_006037.4: c.743C>T p.(Pro248Leu)	NM_006037.4: c.743C>T p.(Pro248Leu)
Parental origin	*de novo*	*de novo*	*de novo*	*de novo*	*de novo*	*de novo*	*de novo*

***In silico* predictions and scores**							

CADD	probably deleterious; 32.0	likely deleterious; 28.2	likely deleterious; 25.1	likely deleterious; 27.3.	likely deleterious; 27.3	likely deleterious; 27.3	likely deleterious; 27.3
PolyPhen-2	probably damaging; 1.000	probably damaging; 1.000	probably damaging; 1.000	probably damaging; 1.000	probably damaging; 1.000	probably damaging; 1.000	probably damaging; 1.000
PROVEAN	deleterious; −5.32	deleterious; −6.21	deleterious; −7.10	deleterious; −8.87	deleterious; −8.87	deleterious; −8.87	deleterious; −8.87
SIFT	damaging; 0.000	damaging; 0.000	damaging; 0.003	damaging; 0.001	damaging; 0.001	damaging; 0.001	damaging; 0.001

**Development**							

Age at walking independently	not achieved	not achieved	18 months	not achieved	not achieved	not achieved; walks with assistance	not achieved; walks with assistance
Education		special school	special school from 7 years	special school		kindergarten with special education classes	special school
Speech development	non-verbal	non-verbal; limited use of Makaton	fluent; first words at 18 months	few single words	non-verbal	single words only	few single words

**Neurology**							

Seizures	infantile spasms; multiple seizure types non-responsive to medication	no seizures	generalized, with multiple seizure types and drop attacks from 11 years	generalized tonic-clonic from 9 years		no seizures	no seizures
Central hypotonia	+	+	+	+	+	+	+
Movement disorder		+		+		−	
MRI brain	cerebral atrophy; brainstem and cerebellar atrophy; abnormal signal within the gray matter nuclei	some cerebellar atrophy; subtle symmetrical signal abnormality of dorsal pons and midbrain	normal	generalized paucity of cerebral and cerebellar bulk of brain matter	slight enlargement of the lateral and 3^rd^ ventricles; prominent extra-axial spaces	thin corpus collosum; nonspecific minor ventriculomegaly; cerebral underdevelopment	normal

**Facial features**							

Hypertelorism		+	+		+	−	+
Full lower lip		+	+	+	+	−	+
Widely spaced teeth		+		+	+	−	+
Frontal upsweep of hair	+	−	+	+	+	−	−
Long palpebral fissures		−	+	+	+	+	+
Large ears		−	+	+		−	+

**Growth parameters**							

Height centile		20^th^	85^th^	3^rd^ at 5 years	99^th^	12^th^	5^th^
Weight centile		9^th^–25^th^	99^th^	2^nd^ at 8 years	15^th^	17^th^	25^th^
Head circumference centile		99^th^	84^th^	29^th^ at 5 years	51^st^	24^th^	50^th^

**Additional features**							

Feeding difficulties	swallowing difficulties; gastrostomy-fed	poor suck, needs pureed food		swallowing difficulties, soft food, thickened fluids		slow bite and chewing of food; oral aversion	+
Drooling		+	+, preschool years	+	+	+	+
Sleep disturbance		+	+	+		−	
Hyperextensibility of finger joints			+	+		−	+
Kyphosis/scoliosis			progressive thoracolumbar scoliosis	kyphosis	scoliosis	postural thoracolumbar kyphosis	scoliosis
Hip anomalies		bilateral femoral head subluxation at 5 years	congenital hip dislocation	congenital hip dislocation		mild lateral subluxation of right hip	
Delayed closure of anterior fontanelle		+		+		−	
Visual problems	absence of tracking		astigmatism	hypermetropia		intermittent esotropia; hyperopia; astigmatism; amblyopia	
Other	apnea; hyperpigmented macule	hydronephrosis; delayed dentition; pectus carinatum; patent ductus arteriosus	severe nut allergy	bilateral talipes equinovarus		asymmetric chest wall; delayed dentition; undescended testis	

Note: CADD (Combined Annotation Dependent Depletion) results show classification and Phred scores; PolyPhen-2 (Polymorphism Phenotyping v2) results show classification and score; PROVEAN (Protein Variation Effect Analyzer) and SIFT (Sorting Intolerant From Tolerant) threshold scores for binary classification were −2.50 and 0.05, respectively; + (plus) denotes feature present; − (minus) denotes feature absent; blank cells indicate data not available, not applicable, or not measured.

### Genetic analysis and sequencing

The molecular methods and bioinformatic pipeline for whole-exome sequencing used in the DDD Study have been previously described.^20^ Individuals 1, 5, 6, and 7 were evaluated by clinical whole-exome sequencing as previously described.^21^ All HDAC4 variants (NM_006037.4) were confirmed by Sanger sequencing.

### *In silico* analysis

To generate sequence logos for protein kinase substrate specificity, the curated dataset of protein kinase substrate sequences was downloaded from the PhosphoSitePlus database and individually filtered for targets of human protein kinases TAK1, MARK2, GSK3A, and GSK3B. Sequence logos were generated with the PhosphoSitePlus Sequence Logo tool using all substrate sequences for each enzyme (TAK1, n = 36; MARK2, n = 31; GSK3A, n = 81; GSK3B, n = 401). Protein hydrophobicity was predicted using the ExPASy ProtScale server; data were calculated in 9-residue windows using the Kyte & Doolittle scale.

### Expression of native and variant HDAC4 and protein interaction assay

Plasmid vectors for expression of epitope-tagged HDAC4 (FLAG-tagged) and 14-3-3β (hemagglutinin [HA]-tagged) have been described previously.^7^^,^^19^ The p.Thr244Lys and p.Glu247Gly variants were created in the FLAG-HDAC4 plasmid by mutagenesis PCR using *PfuUltra* High-Fidelity DNA Polymerase AD (Agilent Technologies). Protein-protein interaction assays were performed by co-immunoprecipitation, as previously described.^19^ Briefly, HEK293 cells were transfected with expression plasmids; at ∼48 h post-transfection, cells were harvested and lysed and cleared extracts used in co-immunoprecipitation assays with antibody directed against the FLAG tag. After scanning, western blots were quantified using ImageJ image processing software; 14-3-3β binding was calculated relative to HDAC4 and 14-3-3β loading controls.

## Results

The clinical features of all individuals are summarized in Table 1, and positions of missense variants within the major 14-3-3 binding site shown in Figure 1A. Further details are provided in the Supplemental notes. All individuals presented with delayed developmental milestones/ID and hypotonia. All individuals of school age have attended special school and have significant ID, though individual 3 had milder cognitive, motor, and speech difficulties in comparison with the rest of the cohort. Individual 1 presented with infantile spasms and continues to have seizures that are non-responsive to medication. Both individual 3 and individual 4 developed generalized seizures in mid-childhood, which have also been difficult to control despite the use of multiple anticonvulsants. Individual 4 has a movement disorder and had hand stereotypies, most noticeable in early childhood. Individual 2 is also described as having dystonic limb movements. Three individuals were reported as suffering from sleep disturbance. None was reported as showing autistic features. Variable and non-specific changes were observed in brain magnetic resonance imaging (MRI) scans in five individuals.

**Figure 2:**
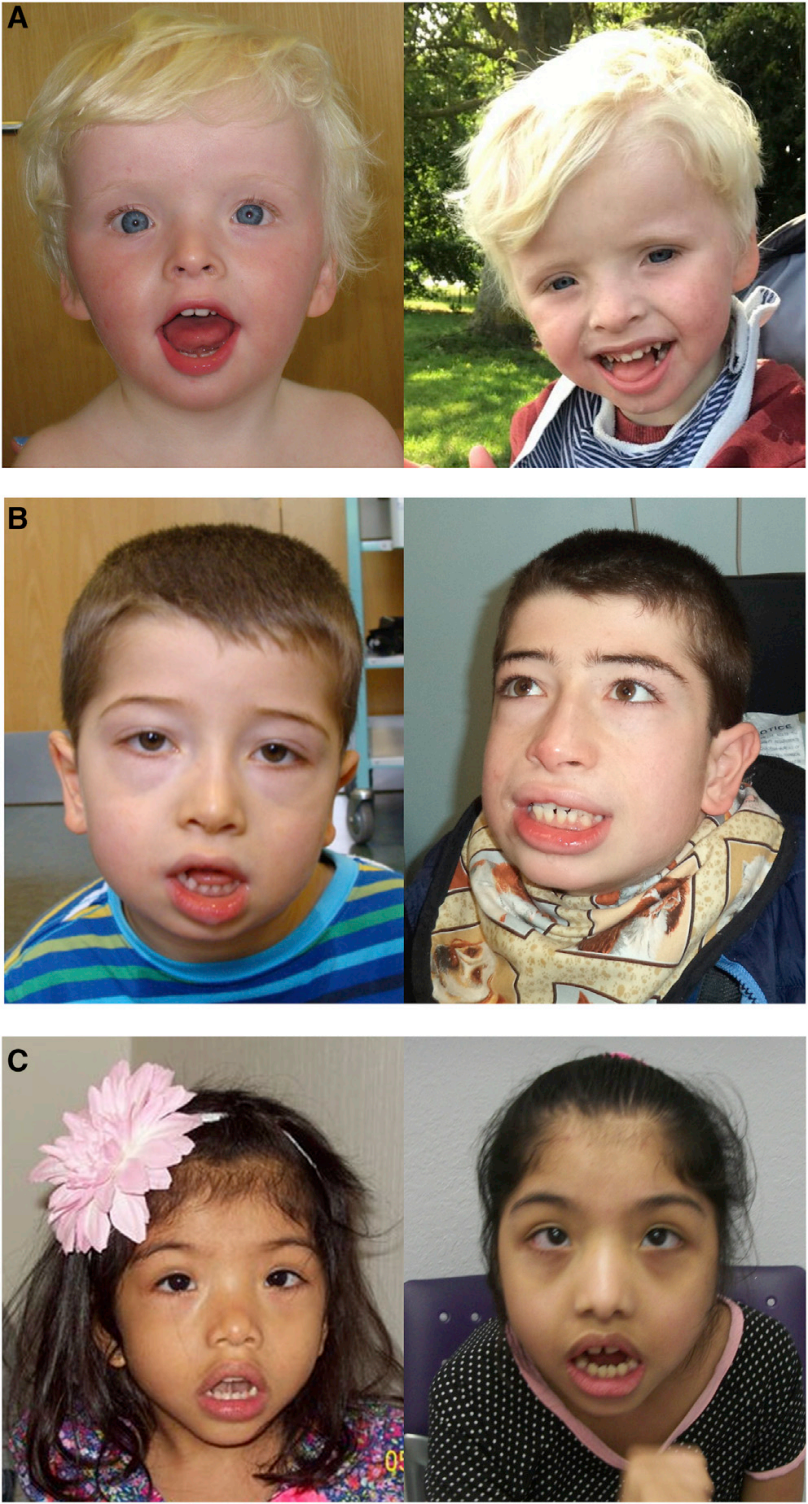
Individuals with *HDAC4* missense variants (A) Individual 2 at ages 2 (left) and 5 years (right). (B) Individual 4 at ages 4 (left) and 14 years (right). He has distinctive facial features with a full lower lip, widely spaced teeth, large ears, straight eyebrows, a frontal upsweep of hair, and relatively long palpebral fissures. (C) Individual 7 at ages 3 (left) and 10 years (right).

Facial appearance was distinctive, with the following features seen in multiple individuals: hypertelorism, a full lower lip, long palpebral fissures, frontal upsweep of hair, widely spaced teeth, and large ears (Figure 2). There is a history of delayed closure of the anterior fontanelle in individuals 2 and 4. Four have had hip dislocation/subluxation and five have scoliosis/kyphosis. Three individuals also have hypermobility, with striking joint laxity of the fingers. Significant drooling in early childhood was a common feature and, where noted, has persisted in all but individual 3. Apart from a relatively large head size in individual 2, growth parameters were generally unremarkable. None of the individuals had hand or foot anomalies consistent with brachydactyly type E.

All *HDAC4* missense variants identified in these individuals lie within the 14-3-3 binding site spanning residues 242–248 of HDAC4 (RKTApSEP, where pSer246 is the phosphorylated residue), with residue Pro248 being affected in 5 individuals. All residues within this motif are highly conserved (Figure 1B), and no missense variants have been observed at any of these positions in the gnomAD database. An analysis of known 14-3-3 binding sites has shown that glycine is not tolerated at the +1 position relative to the phosphorylated residue,^22^ suggesting that the p.Glu247Gly substitution is likely to impair or abolish interaction with 14-3-3. Furthermore, the presence of alanine at the +2 position was also under-represented in known 14-3-3 sites, while the most frequently observed amino acid at this position was proline, suggesting that both the p.Pro248Ala and p.Pro248Leu variants may also be deleterious toward 14-3-3 binding. To further test the predicted effects of these variants, we scanned native and variant HDAC4 sequences using the 14-3-3-Pred server, which uses three independent algorithms to derive a consensus score for each predicted site.^23^ For the site spanning residues 242–248, the p.Glu247Gly variant resulted in a modest decrease in the consensus score to 88.6% of that of the native sequence, while the p.Pro248Ala and p.Pro248Leu variants had a stronger effect, with scores of only 45.9% and 44.5%, respectively, compared to native. The stronger effect of substitutions at Pro248 is consistent with observations that proline plays an important structural role at the +2 position of the 14-3-3 binding motif.^24^ The p.Thr244Lys variant caused only a marginal decrease in the score predicted by 14-3-3-Pred (94.2% compared to native HDAC4); however, while the positively charged arginine commonly occurs at the −2 position, a previous study had concluded that lysine was not tolerated at this position,^22^ suggesting that this variant is also likely to be deleterious for 14-3-3 binding.

**Figure 3:**
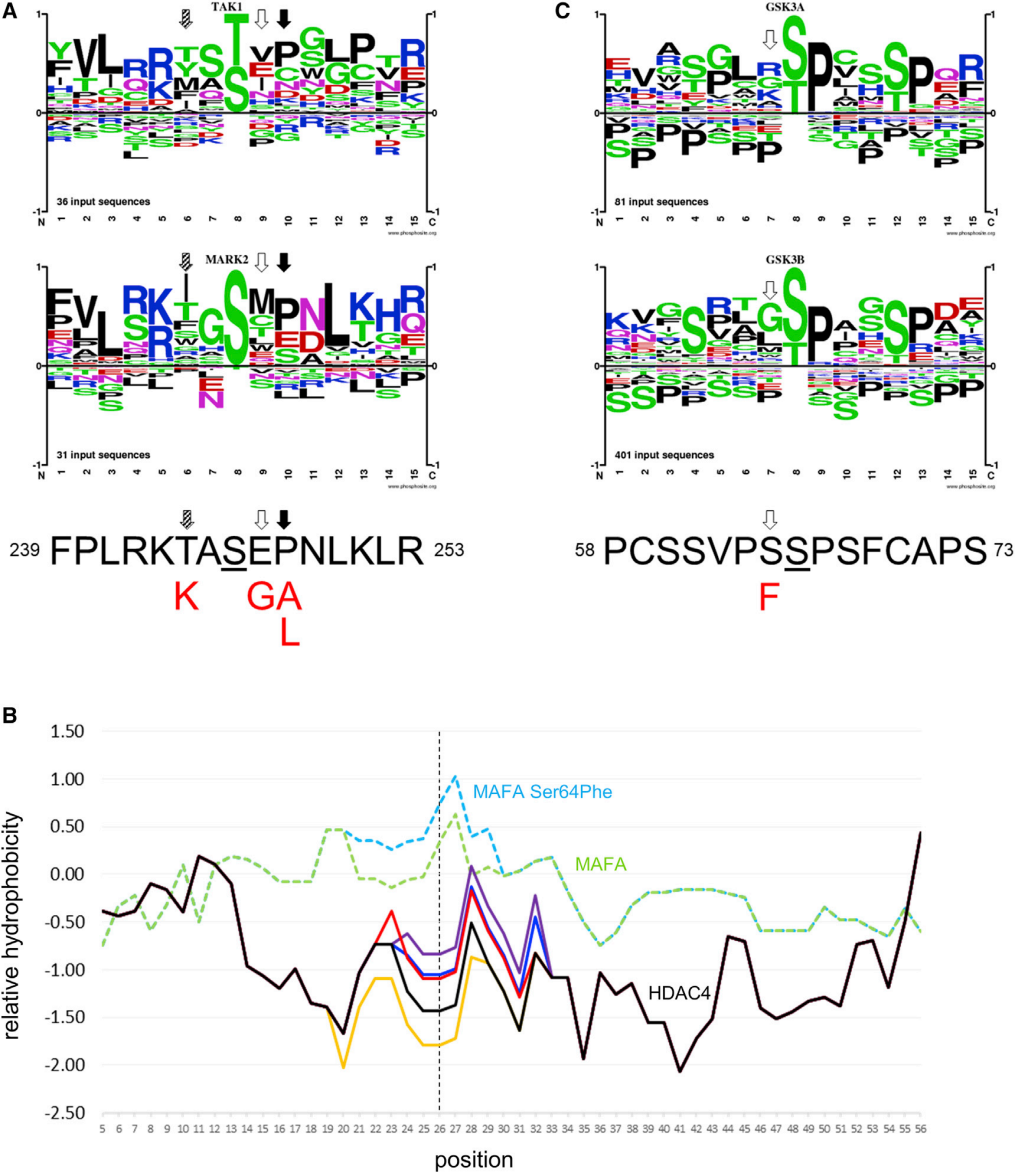
*In silico* analysis of HDAC4 variants (A) Upper panels show sequence logos indicating substrate specificity of TAK1 and MARK2 as indicated; color indicates sidechain properties (blue, positive; red, negative; magenta, neutral; green, polar; black, hydrophobic), and all sequences are centered on the phosphorylated serine or threonine residue at position 8 of the logo. Below these is shown the sequence of HDAC4 residues 239–253 (black font; the phosphorylated serine, Ser246, is underlined); residues Glu247 and Pro248 are indicated by open and filled arrows, respectively, and these align to positions 9 and 10 of substrate logos (or +1 and +2 relative to the phosphorylated serine) as marked; residue Thr244 is marked by a hatched arrow and lies at position 6 of the logo (−2 relative to Ser246); variants observed at these positions are shown in red font below the sequence. (B) Relative hydrophobicity is shown for residues 221–280 of HDAC4 (solid black line) and for variants p.Glu247Gly, p.Pro248Ala, p.Pro248Leu, and p.Thr244Lys (solid red, blue, purple, and gold lines, respectively); broken lines show hydrophobicity of residues 20–79 of MAFA (green) and MAFA variant Ser64Phe (light blue); in all cases, traces are aligned to show the phosphorylated residue (HDAC4 Ser246; MAFA Ser65) at position 26 of the analysis, as indicated by the vertical broken line. (C) Similar to (A), showing substrate specificity logos for GSK3A (top) and GSK3B (center); the sequence below shows residues 58–73 of MAFA, with the phosphorylated serine (Ser65) underlined; the position of the p.Ser64Phe variant is shown by an open arrow in all parts.

Since 14-3-3 binding is known to be enhanced by phosphorylation, we examined *in silico* the potential effect of variants on the substrate specificity for the protein kinases TAK1 and MARK2, which have been reported to phosphorylate Ser246 *in vivo* (PhosphoSitePlus database, accessed May 12, 2020).^25^ Like most protein kinases, these enzymes have somewhat relaxed and overlapping specificities; however, inspection of the sequence logos for both suggested that in all individuals the observed HDAC4 variants were likely to result in a less-preferred substrate for phosphorylation (Figure 3A). The predicted effect was likely to be strongest for variants at Pro248, where proline is the preferred residue at the +2 position for both kinases, and for the p.Thr244Lys variant, where threonine is the first- or second-most preferred amino acid at the −2 position for TAK1 or MARK2, respectively.

The 14-3-3 site at residues 242–248 lies at the N-terminal of a region of predicted intrinsic disorder in HDAC4. Such regions are common in transcription factors and other regulators of gene expression and are over-represented in so-called short linear motifs (SLiMs), which may act as targets for post-translational modification or to confer transient interactions with binding partners, the 14-3-3 motif being one such example of the latter category.^26^^,^^27^ The inherent lack of stable structure in disordered regions allows SLiMs to be exposed to solvent and, thus, accessible for binding; conversely, variants that reduce accessibility or increase hydrophobicity may reduce the functionality of such motifs. Plotting the hydrophobicity for residues 221–280 of native and variant HDAC4 showed that variants at Glu247 and Pro248 resulted in an increase in hydrophobicity at the position of Ser246, which could potentially reduce its accessibility to protein kinases (Figure 3B). Interestingly, the magnitude of this effect was similar to that of the MAF BZIP Transcription Factor A (MAFA) variant p.Ser64Phe in comparison to its native sequence. MAFA Ser64 lies in the intrinsically disordered N-terminal region of the protein and adjacent to Ser65, which is the target of phosphorylation by glycogen synthase kinase 3 (GSK3). Functional analysis had revealed that the p.Ser64Phe variant resulted in reduced phosphorylation of MAFA Ser65 *in vivo*,^28^ and as the serine-to-phenylalanine substitution was not predicted to have a significant effect on the substrate specificity of either GSK3A or GSK3B (Figure 3C), it seems likely that the reduced phosphorylation was a consequence of increased hydrophobicity at the target site. In contrast to the effects of other variants, the p.Thr244Lys variant resulted in decreased hydrophobicity at Ser246, suggesting that, in this case, accessibility of this site to protein kinases would likely be unimpaired in comparison to that in native HDAC4.

**Figure 4:**
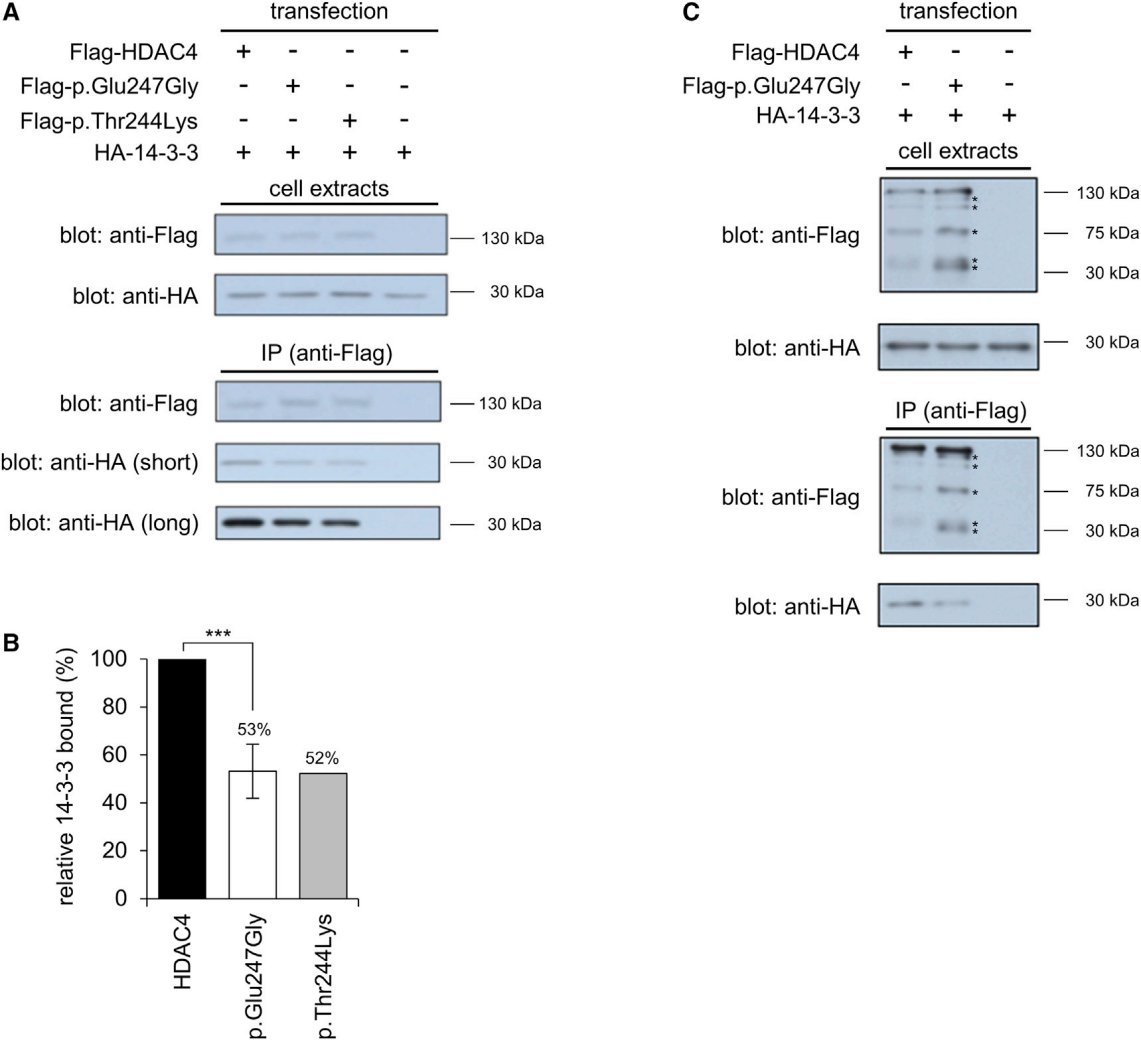
Variants p.Glu247Gly and p.Thr244Lys have reduced affinity for 14-3-3β (A) HEK293 cells were transfected with expression plasmids for wild-type HDAC4, or the p.Thr244Lys or p.Glu247Gly variants as shown, then extracts subjected to immunoprecipitation (IP) with anti-FLAG antibody. Upper panels show western blots of cell extracts using either anti-FLAG (to detect HDAC4) or anti-HA (to detect 14-3-3β); lower panels show western blots of IP samples using the same antibodies, showing both short and long exposures of the anti-HA blot. Data shown for variant p.Glu247Gly are representative of five independent experiments; the p.Thr244Lys variant was tested in a single experiment, as shown here. (B) Relative binding of 14-3-3β by the p.Glu247Gly variant was quantified from five independent experiments; error bars show standard deviation from the mean value; statistical significance was calculated using Student’s t test, ***p < 0.001; data for p.Thr244Lys were derived from the single experiment shown in (A). (C) As in (A), showing data from an independent experiment after transfection of expression plasmids for wild-type HDAC4 or the p.Glu247Gly variant only; extended regions of the anti-HA blots are shown to demonstrate C-terminal-truncated products of HDAC4 fragmentation, indicated by asterisks (*).

To test the effects predicted *in silico* on the binding of 14-3-3, we co-expressed 14-3-3β with either native or variant HDAC4 in HEK293 cells. Co-immunoprecipitation assays carried out on cell extracts indicated that both the p.Glu247Gly and p.Thr244Lys variants bound 14-3-3β with approximately two-fold reduced affinity compared to native HDAC4 (Figures 4A and 4B), consistent with the hypothesis that these variants result in increased nuclear HDAC4 and a gain-of-function phenotype. As has been previously observed,^19^ both native and variant HDAC4 were subject to fragmentation within the cell; interestingly, the proportion of C-terminal truncated fragments was higher for the p.Glu247Gly variant compared to native HDAC4 (Figure 4C), while similar results were observed for the p.Thr244Lys variant (not shown), and this may have further implications for the effects of these variants *in vivo*.

## Discussion

All variants reported here lie within a motif that is known to mediate interaction with 14-3-3 proteins in a phosphorylation-dependent manner and that plays a major role in regulating the nucleocytoplasmic shuttling of HDAC4. *In silico* analyses predicted that all variants were likely to result in reduced binding of 14-3-3 proteins, whether by decreased affinity of 14-3-3 for the variant motif, reduced phosphorylation of Ser246, or a combination of both effects. Taken together, these data suggest that all variants may result in reduced sequestration of HDAC4 in the cytoplasm by 14-3-3 proteins and increased levels of nuclear HDAC4. Consistent with this hypothesis, co-immunoprecipitation assays showed the p.Glu247Gly and p.Thr244Lys variants bound 14-3-3β with reduced affinity compared to native HDAC4. As such, we propose that these variants result in a novel gain-of-function effect, leading to increased nuclear activity of HDAC4, and thus differentiating both the molecular mechanism and the outcome of these variants from that of previously reported loss-of-function alleles of *HDAC4* that result in haploinsufficiency. We note that the effect of the variants on 14-3-3 binding was only partial, suggesting that any increase in nuclear HDAC4 activity might be limited. However, it is likely that HDAC4 activity is tightly controlled in the cell, and circumstantial evidence suggests that substantial increases in this activity may be incompatible with life: first, no whole-gene duplications, which might give rise to increased expression through higher gene dosage, have been reported at the *HDAC4* locus; second, attempts to generate mice carrying the constitutive nuclear 3SA mutant of HDAC4, in which the serine residues of the three major 14-3-3 sites (Ser246, Ser467, and Ser632) were mutated to alanine, were unsuccessful.^29^ It is unclear, therefore, what level of increased nuclear HDAC4 activity can be tolerated during normal development, and it is entirely feasible that pathological changes might result from only modest changes in HDAC4-regulated gene expression.

Consistent with a different mechanism of action, the phenotype of individuals reported here differs from that reported in BDMR.^14^, ^15^, ^16^, ^17^ Individuals shared facial dysmorphic features that were distinct from the broad face, brachycephaly, and broad, upturned nose described in BDMR. None of the individuals reported here had autism, obesity, or brachydactyly type E. Cognitive disability, seizures, and sleep disturbance are reported in BDMR; however, the DD/ID in our individuals was more significant. The individuals reported here also had features not typically seen in individuals with 2q37 deletion or loss-of-function variants in *HDAC4*, including swallowing difficulties and/or drooling, congenital hip dislocation, progressive kyphoscoliosis, and delayed closure of the anterior fontanelle.

Increased nuclear activity of HDAC4 would be expected to result in reduced activity of RUNX2 and MEF2C. In support of this, Vega et al.^11^ have previously noted that overexpression of HDAC4 in proliferating mouse chondrocytes inhibited their differentiation, thus resembling the effects of *Runx2* loss of function. We noted some non-specific phenotypic overlap between our individuals and those with variants in *MEF2C* (MIM: 613443), including DD/ID, stereotypic movements, epilepsy, and variable MRI brain scan anomalies. In addition, *RUNX2* loss-of-function alleles cause cleidocranial dysplasia (CDD [MIM: 119600]), a feature of which is delayed closure of the anterior fontanelle, which was reported in two of the individuals described here. Moreover, although there was limited overlap with the widespread, characteristic skeletal defects seen in CDD, at least 6/7 individuals in this report displayed dental anomalies, hypertelorism, and/or defects of the hip joints, all of which have been observed in individuals with pathogenic *RUNX2* variants.^30^, ^31^, ^32^ Whether the decreased interaction with 14-3-3 does indeed lead to decreased RUNX2 and MEF2C signaling as a result of increased nuclear HDAC4 activity remains to be determined; furthermore, the presence of unique features in the individuals reported here, which are not observed in either *RUNX2* or *MEF2C* loss-of-function phenotypes, suggests the possible involvement of other, as-yet-unidentified pathways downstream of HDAC4. Nevertheless, the observation of four different missense variants, all of which affect the same functional motif in HDAC4 and arose *de novo* in seven unrelated individuals, provides very strong genetic evidence that these variants are indeed causative for the phenotype observed.

HDAC4 has been shown to specifically repress the expression of genes required for synaptic function and neuronal plasticity, and this expression was reduced further by the constitutive nuclear 3SA mutant of HDAC4.^29^ Interestingly, a similar effect was observed for the BDMR variant c.2399_2400insC,^15^ which when expressed from a cDNA construct results in truncation of HDAC4 after amino acid Gly801 and is constitutive nuclear due to loss of the C-terminal nuclear export signal, although detailed transcriptomic analysis was not performed for this variant.^29^ This truncated variant differs from the 3SA mutant and those in the individuals reported here in that it lacks an intact deacetylase domain and therefore will be catalytically inactive against acetylated histones or other target proteins, but it retains sequences required for binding to MEF2. This suggests that some of the specific neurological features observed in our individuals may be due to increased binding of MEF2 by HDAC4 rather than due to altered nuclear deacetylase activity, although these mechanisms are not necessarily exclusive and further work is required to distinguish between the downstream consequences of these two pathways. Interestingly, the fragmentation of HDAC4 that occurs *in vivo* also generates truncated proteins that have been reported to retain the ability to repress MEF2C activity,^33^ and these include the product of caspase-mediated cleavage at Asp289, which likely corresponds to the band at ∼33 kDa in our data (Figure 4C). As both the p.Glu247Gly and p.Thr244Lys variants showed increased fragmentation during transient expression, this raises the possibility that increased repression of MEF2C by these fragments might play a role in the proposed gain-of-function effect of the variants, in addition to that conferred by reduced 14-3-3 binding and independent of deacetylase activity. Moreover, it is possible that an increase in the level of these truncated proteins, which persist in the nucleus due to loss of the C-terminal nuclear export signal, may amplify the effects of reduced 14-3-3 binding.

Gain-of-function variants in *HDAC4* raise the possibility of therapeutic intervention by use of HDAC inhibitors, although whether such treatment could halt or reverse developmental problems is open to question. While a number of HDAC inhibitors are available for pharmacological use, class IIa HDACs are in fact poor responders to these drugs due to the lower deacetylase activity of these enzymes compared to other HDACs.^34^ Effective therapy would therefore require development of inhibitors with specificity for class IIa enzymes (and preferably HDAC4 alone); a recent study reported the development of improved cyclopropane hydroxamic acid derivatives for inhibition of HDAC4 deacetylase activity,^35^ while an alternative approach has been to target the interaction with MEF2.^36^

In summary, we present evidence that variants within the key 14-3-3 binding motif spanning residues 242–248 of HDAC4 cause a novel phenotype, as a result of reduced 14-3-3 binding, which we hypothesize leads to reduced cytoplasmic retention and thus an increase in the nuclear activity of HDAC4. This phenotype includes significant DD/ID, seizures, distinctive facial features, scoliosis, delayed closure of the anterior fontanelle, and non-specific brain MRI anomalies; thus, it is distinct from the BDMR phenotype previously reported in individuals carrying *HDAC4* loss-of-function alleles. Identification of additional individuals with variants within this region of *HDAC4* will enable further genotype-phenotype correlation and understanding of the clinical spectrum in similar individuals.

## Declaration of Interests

The authors declare no competing interests.

## Web resources

14-3-3-Pred server, http://www.compbio.dundee.ac.uk/1433pred
ClinVar, https://www.ncbi.nlm.nih.gov/clinvar/
DECIPHER, https://decipher.sanger.ac.uk/
ExPASy ProtScale server, https://web.expasy.org/protscale/
Genome Aggregation Database (gnomAD) v2.1.1, https://gnomad.broadinstitute.org/
ImageJ: Image Processing and Analysis in Java, https://imagej.nih.gov/ij/
OMIM, https://omim.org/
PhosphoSitePlus database, file Kinase_Substrate_Dataset.gz, https://www.phosphosite.org
UniProtKB, https://www.uniprot.org/

## Supplemental information

Document 1. Supplemental notes

Document 2. Article plus supplemental information

## Footnotes

Supplemental Information can be found online at https://doi.org/10.1016/j.xhgg.2020.100015.

## References

[1] Verdin E., Dequiedt F., Kasler H.G. (2003). Class II histone deacetylases: versatile regulators. Trends Genet..

[2] Yang X.J., Seto E. (2008). The Rpd3/Hda1 family of lysine deacetylases: from bacteria and yeast to mice and men. Nat. Rev. Mol. Cell Biol..

[3] Haberland M., Montgomery R.L., Olson E.N. (2009). The many roles of histone deacetylases in development and physiology: implications for disease and therapy. Nat. Rev. Genet..

[4] Grozinger C.M., Hassig C.A., Schreiber S.L. (1999). Three proteins define a class of human histone deacetylases related to yeast Hda1p. Proc. Natl. Acad. Sci. USA.

[5] Fischle W., Emiliani S., Hendzel M.J., Nagase T., Nomura N., Voelter W., Verdin E. (1999). A new family of human histone deacetylases related to *Saccharomyces cerevisiae* HDA1p. J. Biol. Chem..

[6] Miska E.A., Karlsson C., Langley E., Nielsen S.J., Pines J., Kouzarides T. (1999). HDAC4 deacetylase associates with and represses the MEF2 transcription factor. EMBO J..

[7] Wang A.H., Bertos N.R., Vezmar M., Pelletier N., Crosato M., Heng H.H., Th’ng J., Han J., Yang X.J. (1999). HDAC4, a human histone deacetylase related to yeast HDA1, is a transcriptional corepressor. Mol. Cell. Biol..

[8] Lemercier C., Verdel A., Galloo B., Curtet S., Brocard M.P., Khochbin S. (2000). mHDA1/HDAC5 histone deacetylase interacts with and represses MEF2A transcriptional activity. J. Biol. Chem..

[9] Kao H.Y., Downes M., Ordentlich P., Evans R.M. (2000). Isolation of a novel histone deacetylase reveals that class I and class II deacetylases promote SMRT-mediated repression. Genes Dev..

[10] McKinsey T.A., Zhang C.L., Lu J., Olson E.N. (2000). Signal-dependent nuclear export of a histone deacetylase regulates muscle differentiation. Nature.

[11] Vega R.B., Matsuda K., Oh J., Barbosa A.C., Yang X., Meadows E., McAnally J., Pomajzl C., Shelton J.M., Richardson J.A. (2004). Histone deacetylase 4 controls chondrocyte hypertrophy during skeletogenesis. Cell.

[12] Clocchiatti A., Di Giorgio E., Viviani G., Streuli C., Sgorbissa A., Picco R., Cutano V., Brancolini C. (2015). The MEF2-HDAC axis controls proliferation of mammary epithelial cells and acini formation *in vitro*. J. Cell Sci..

[13] Williams S.R., Aldred M.A., Der Kaloustian V.M., Halal F., Gowans G., McLeod D.R., Zondag S., Toriello H.V., Magenis R.E., Elsea S.H. (2010). Haploinsufficiency of *HDAC4* causes brachydactyly mental retardation syndrome, with brachydactyly type E, developmental delays, and behavioral problems. Am. J. Hum. Genet..

[14] Wheeler P.G., Huang D., Dai Z. (2014). Haploinsufficiency of *HDAC4* does not cause intellectual disability in all affected individuals. Am. J. Med. Genet. A..

[15] Le T.N., Williams S.R., Alaimo J.T., Elsea S.H. (2019). Genotype and phenotype correlation in 103 individuals with 2q37 deletion syndrome reveals incomplete penetrance and supports HDAC4 as the primary genetic contributor. Am. J. Med. Genet. A..

[16] Villavicencio-Lorini P., Klopocki E., Trimborn M., Koll R., Mundlos S., Horn D. (2013). Phenotypic variant of Brachydactyly-mental retardation syndrome in a family with an inherited interstitial 2q37.3 microdeletion including *HDAC4*. Eur. J. Hum. Genet..

[17] Karczewski K.J., Francioli L.C., Tiao G., Cummings B.B., Alföldi J., Wang Q., Collins R.L., Laricchia K.M., Ganna A., Birnbaum D.P. (2020). The mutational constraint spectrum quantified from variation in 141,456 humans. Nature.

[18] Grozinger C.M., Schreiber S.L. (2000). Regulation of histone deacetylase 4 and 5 and transcriptional activity by 14-3-3-dependent cellular localization. Proc. Natl. Acad. Sci. USA.

[19] Wang A.H., Kruhlak M.J., Wu J., Bertos N.R., Vezmar M., Posner B.I., Bazett-Jones D.P., Yang X.J. (2000). Regulation of histone deacetylase 4 by binding of 14-3-3 proteins. Mol. Cell. Biol..

[20] Wright C.F., Fitzgerald T.W., Jones W.D., Clayton S., McRae J.F., van Kogelenberg M., King D.A., Ambridge K., Barrett D.M., Bayzetinova T. (2015). Genetic diagnosis of developmental disorders in the DDD study: a scalable analysis of genome-wide research data. Lancet.

[21] Yang Y., Muzny D.M., Reid J.G., Bainbridge M.N., Willis A., Ward P.A., Braxton A., Beuten J., Xia F., Niu Z. (2013). Clinical whole-exome sequencing for the diagnosis of mendelian disorders. N. Engl. J. Med..

[22] Panni S., Montecchi-Palazzi L., Kiemer L., Cabibbo A., Paoluzi S., Santonico E., Landgraf C., Volkmer-Engert R., Bachi A., Castagnoli L., Cesareni G. (2011). Combining peptide recognition specificity and context information for the prediction of the 14-3-3-mediated interactome in *S. cerevisiae* and *H. sapiens*. Proteomics.

[23] Madeira F., Tinti M., Murugesan G., Berrett E., Stafford M., Toth R., Cole C., MacKintosh C., Barton G.J. (2015). 14-3-3-Pred: improved methods to predict 14-3-3-binding phosphopeptides. Bioinformatics.

[24] Rittinger K., Budman J., Xu J., Volinia S., Cantley L.C., Smerdon S.J., Gamblin S.J., Yaffe M.B. (1999). Structural analysis of 14-3-3 phosphopeptide complexes identifies a dual role for the nuclear export signal of 14-3-3 in ligand binding. Mol. Cell.

[25] Dequiedt F., Martin M., Von Blume J., Vertommen D., Lecomte E., Mari N., Heinen M.F., Bachmann M., Twizere J.C., Huang M.C. (2006). New role for hPar-1 kinases EMK and C-TAK1 in regulating localization and activity of class IIa histone deacetylases. Mol. Cell. Biol..

[26] Ren S., Uversky V.N., Chen Z., Dunker A.K., Obradovic Z. (2008). Short Linear Motifs recognized by SH2, SH3 and Ser/Thr Kinase domains are conserved in disordered protein regions. BMC Genomics.

[27] Uversky V.N., Oldfield C.J., Dunker A.K. (2008). Intrinsically disordered proteins in human diseases: introducing the D2 concept. Annu. Rev. Biophys..

[28] Iacovazzo D., Flanagan S.E., Walker E., Quezado R., de Sousa Barros F.A., Caswell R., Johnson M.B., Wakeling M., Brändle M., Guo M. (2018). *MAFA* missense mutation causes familial insulinomatosis and diabetes mellitus. Proc. Natl. Acad. Sci. USA.

[29] Sando R., Gounko N., Pieraut S., Liao L., Yates J., Maximov A. (2012). HDAC4 governs a transcriptional program essential for synaptic plasticity and memory. Cell.

[30] Bedeschi M.F., Bonarrigo F., Manzoni F., Milani D., Piemontese M.R., Guez S., Esposito S. (2014). Ehlers-Danlos syndrome versus cleidocranial dysplasia. Ital. J. Pediatr..

[31] Jaruga A., Hordyjewska E., Kandzierski G., Tylzanowski P. (2016). Cleidocranial dysplasia and RUNX2-clinical phenotype-genotype correlation. Clin. Genet..

[32] Hordyjewska E., Jaruga A., Kandzierski G., Tylzanowski P. (2017). Novel mutation of the *RUNX2* gene in patients with cleidocranial dysplasia. Mol. Syndromol..

[33] Paroni G., Mizzau M., Henderson C., Del Sal G., Schneider C., Brancolini C. (2004). Caspase-dependent regulation of histone deacetylase 4 nuclear-cytoplasmic shuttling promotes apoptosis. Mol. Biol. Cell.

[34] Bradner J.E., West N., Grachan M.L., Greenberg E.F., Haggarty S.J., Warnow T., Mazitschek R. (2010). Chemical phylogenetics of histone deacetylases. Nat. Chem. Biol..

[35] Stoddard S.V., Dodson K., Adams K., Watkins D.L. (2019). *In silico* design of novel histone deacetylase 4 inhibitors: design guidelines for improved binding affinity. Int. J. Mol. Sci..

[36] Jayathilaka N., Han A., Gaffney K.J., Dey R., Jarusiewicz J.A., Noridomi K., Philips M.A., Lei X., He J., Ye J. (2012). Inhibition of the function of class IIa HDACs by blocking their interaction with MEF2. Nucleic Acids Res..

