# Supplementary material for: Missense substitutions at a conserved 14-3-3 binding site in HDAC4 cause a novel intellectual disability syndrome
Source: HGG Adv. 2020 Nov 21;2(1):100015. doi: 10.1016/j.xhgg.2020.100015 (PMC7841527; doi:10.1016/j.xhgg.2020.100015)
Supplement: Document 1. Supplemental notes [file mmc1.pdf]

XHGG, Volume *2*

Supplemental Information

Missense substitutions at a conserved 14-3-3 binding site in HDAC4 cause a novel intellectual disability syndrome

Emma Wakeling, Meriel McEntagart, Michael Bruccoleri, Charles Shaw-Smith, Karen L. Stals, Matthew Wakeling, Angela Barnicoat, Clare Beesley, DDD Study, Andrea K. Hanson-Kahn, Mary Kukolich, David A. Stevenson, Philippe M. Campeau, Sian Ellard, Sarah H. Elsea, Xiang-Jiao Yang, and Richard C. Caswell

Supplemental information: case histories

Case 1

A 2 year old girl evaluated in the paediatric intensive care unit for management of floppy airways and currently on oxygen in preparation for tracheostomy tube placement. She had airway problems from birth, with apnoea since 3 weeks of age. She had no control of head movement at 4 months of age. Infantile spasms began at 8 months of age; seizures began shortly thereafter. Seizures are currently non-responsive to therapy.

General physical exam at 22 months was unremarkable. Eyes appeared normal and there was an upsweep of the hair and scalp. There was no evidence for scoliosis; however, a full exam could not be completed. Plantar responses were downward bilaterally, and deep tendon reflexes were not increased. Recent retinal exam was normal. Gastrostomy feeding tube (G-tube) placement reportedly improved eye contact and alertness; however, her eyes currently do not track.

Brain magnetic resonance imaging (MRI) at 24 months of age showed cerebral atrophy and to a lesser degree, brainstem and cerebellar atrophy, with abnormal signal within the grey matter nuclei, suspicious for a significant neurodegenerative process.

Previous testing performed at around 1 year of age for developmental delay and hypotonia included a normal karyotype (46,XX), normal chromosome microarray, and a muscle biopsy with normal histology and mitochondrial electron transport chain enzyme testing suggestive of mitochondrial proliferation**.**

Case 2

A 6 month old male infant born to non-consanguineous parents presented with global developmental delay and dysmorphism. The pregnancy was complicated by an elevated nuchal translucency measurement of 2.3 mm at 12 weeks and short femurs at 20 weeks. Chorionic villus sampling excluded the common trisomies. He weighed 3.45 kg at term. He was smiling, fixing and following from 8 weeks and could reach and transfer objects at 1 year. At 2 years he was sitting with support but was not rolling or pushing up when prone. At 6 years, he is still non-ambulant and has no language but has some limited use of Makaton. His symbolic understanding and play have not developed. Hearing and vision are normal. His sleep is poor. He has been a poor feeder from infancy, with a weak suck. He weaned onto purees but still does not chew. His first teeth erupted at the age of 1 year. At 2 years of age he had a persistently large anterior fontanelle. He developed bilateral femoral head subluxation at 5 years.

Upon examination at 5 years his occipital frontal head circumference is on the 99th centile, weight is on the 9th-25th centile and length on the 20th centile. He is hyperteloric with a flat nasal bridge and full lower lip. He has a slightly narrow chest with sternal recession. His central tone is low, but deep tendon reflexes are normal.

There were abnormal findings from extensive investigations: brain MRI revealed some subtle symmetrical signal abnormality of the dorsal pons and midbrain suggestive of restricted diffusion and a possible underlying neurometabolic disorder; skeletal survey indicated a

generalised delay in ossification, large anterior fontanelle and a probable abnormal Wormian bone pattern; renal ultrasound revealed unilateral hydronephrosis.

Case 3

A 20 year old girl, born to non-consanguineous parents was referred to the genetic clinic at 5 years for developmental delay. She was born at term by normal delivery, with a birth weight of 3.52 kg and head circumference of 34.5 cm. Apart from meconium-stained liquor there were no neonatal concerns. She had truncal hypotonia, poor head control and her motor milestones were delayed from the outset, sitting at 8 months and walking at 18 months. She said her first words at 18 months but was slow to progress and required speech therapy. She attended a mainstream school to 7 years and then transferred to a special school. She is currently learning life skills. She is able to undress herself but needs help with dressing and cutting up her food. She wears glasses for astigmatism. Her hearing is normal.

From 11 years of age she developed generalised seizures with multiple seizure types. She has been treated with lamotrigine, clobazam, sodium valproate and levetiracetam but continues to have regular drop attacks.

She was treated for bilateral congenital hip dysplasia and in the last year has been diagnosed with progressive thoracolumbar scoliosis. In early childhood she drooled excessively, though this resolved once she reached school age. She also has asthma and a severe nut allergy.

She has distinctive facial features with thick, straight eyebrows, slight frontal upsweep of her hair and a full lower lip. Photographs at 7 years also show hypertelorism, which has resolved. She has marked hypermobility, flat feet and significant joint laxity in her fingers which she cannot actively extend, though there is no camptodactyly.

Chromosomal array and fragile X testing were normal. Her brain MRI scan was also normal.

Case 4

A 14 year old boy born to non-consanguineous parents presented at 16 months with global developmental delay. He was diagnosed antenatally with bilateral talipes equinovarus. The pregnancy was also complicated by cardiac arrhythmia which settled antenatally. He was born at 41+4 weeks gestation by normal vaginal delivery with a birth weight of 3.29 kg. There were no concerns in the neonatal period, and he fed well from birth. However, from early on he was noted to have delayed milestones and central hypotonia. He sat at 16 months but remains unable to walk. When younger he had persistent hand regard and limited grasp of objects abnormal purposeless hand movements but can now grasp objects briefly and finger feed. He understands some familiar phrases with the benefit of environmental context. He makes some vocalisations and has a few single words. He has some understanding of cause and effect and is at an early stage of container play. He remains doubly incontinent. His hearing is normal. He has been prescribed glasses for hypermetropia.

From the age of around 9 years he developed generalised tonic-clonic seizures, which have worsened in the last 2 years. He is currently treated with sodium valproate and levetiracetam. He also has a bilateral whole body motor disorder with fluctuating tone. He drools

excessively and has had swallowing difficulties since 11 years of age, needing soft food and thickened fluids. His talipes was treated shortly after birth with casting and bilateral tenotomies. He is generally hypermobile and has bilateral dislocation of his hips and progressive kyphosis. There is also a history of delayed closure of his anterior fontanelle. His primary dentition erupted at the normal time but some primary teeth have failed to exfoliate.

At 5 years of age, his height and head circumference were recorded on 3rd and 29th centiles, respectively. He has distinctive facial features with a full lower lip, widely spaced teeth, large ears, straight eyebrows, frontal upsweep of hair and relatively long palpebral fissures. He has bilateral fixed talipes equinovarus, fixed flexion at the elbows, an unusual bony configuration of his knees and kyphosis. There is joint laxity in his fingers. He has low truncal tone; his reflexes are normal.

His brain MRI scan showed generalised paucity of cerebral and cerebellar bulk of brain matter but no specific structural abnormalities. Chromosomal array and baseline metabolic tests were normal.

Case 5

A 5 year old boy, born to non-consanguineous parents presented with global developmental delay. He was born at 36 weeks gestation, weighing 2.92 kg. In the neonatal period he suffered from apnoea and had central hypotonia. He sat independently at over 1 year and at the age of 5 years is still not walking and has no recognisable words. He has no history of seizures. He has hypertelorism, a low nasal bridge, widely-spaced teeth, full lips and frontal upsweep of his hair. He has a peri-oral rash, suggestive of drooling. He also has scoliosis and increased lower limb reflexes.

A brain MRI scan showed slight enlargement of the lateral and 3rd ventricles and prominent extra axial spaces.

Case 6

A 2 year old boy was initially referred for developmental delay and re-evaluated at 5 years of age. His early developmental milestones have all been delayed, and he is hypotonic. He currently attends kindergarten with special education classes and has input from occupational therapy and physiotherapy. He has some word approximations and will mimic single words but is not yet signing. He has feeding difficulties characterized by difficulties with biting and chewing food, oral aversion and drooling. He has intermittent esotropia, hyperopia, astigmatism and amblyopia. He had unilateral cryptorchidism. Eruption of his primary dentition was delayed. He has mild lateral subluxation of his right hip. There is no history of seizures to date.

On examination, he has an up-turned nasal tip, long philtrum, micrognathia, high-arched palate and low anterior hairline. He has long eyelashes, bushy eyebrows and a slight lateral extension of the palpebral fissures. His ears are low-set with unusual lobes. He has an asymmetric chest wall with postural thoracolumbar scoliosis. His growth parameters are all in the normal range.

His brain MRI scan shows a thin corpus callosum, nonspecific mild ventriculomegaly and cerebral underdevelopment.

Case 7

A 10 year old female was seen for re-evaluation because of the finding of a missense variant in *HDAC4* on whole exome sequencing. She had initially been referred with developmental delay and dysmorphic features. She was born at 36 weeks gestation. Early developmental milestones were all delayed. At 10 years of age, her growth is relatively normal, although her height is now slightly below the 5th percentile. She can walk with some assistance but can only stand for short periods of time. She is nonverbal and only signs simple words such as "momma". She has had problems with drooling. There is no history of seizures. She has facial dysmorphism with hypertelorism and a somewhat prominent nose with open mouth and full lips.

Brain MRI scan was reported as normal. Microarray showed a small chromosome 5 short arm (5p) duplication which was inherited from her normal mother and thought to be a population variant.
